# Trends of Major Cerebrovascular Procedures After Mechanical Thrombectomy Implementation: A Nationwide Observational Study in Germany (2015–2023)

**DOI:** 10.3390/brainsci16050444

**Published:** 2026-04-23

**Authors:** Sara Hirsch, Karel Kostev, Ali Hammed, Bert Bosche, Marek Molcanyi, Christian Tanislav

**Affiliations:** 1 Department of Geriatrics and Neurology, Diakonie Hospital Jung Stilling Siegen, 57074 Siegen, Germany; sara.hirsch@diakonie-sw.de (S.H.); alihaamed9@gmail.com (A.H.); 2 Epidemiology, IQVIA, 60549 Frankfurt am Main, Germany; karel.kostev@iqvia.com; 3 Department of Neurology, Ev. Hospital Mettmann, 40822 Mettmann, Germany; b.bosche@evk-mettmann.de; 4 Department of Neurology, University Hospital Essen, Medical Faculty, University of Duisburg-Essen, 45147 Essen, Germany; 5 Department of Neurology, Mediclin Hospital Reichshof, 51580 Reichshof-Eckenhagen, Germany; molcanyi77@gmail.com

**Keywords:** stroke, mechanical thrombectomy, decompressive hemicraniectomy, epidemiology, neuroepidemiology

## Abstract

**Background**: New therapies like intravenous thrombolysis (IVT) and mechanical thrombectomy (MT) for acute ischemic stroke (AIS) can significantly impact outcomes and complication rates. This study examines the nationwide impact of MT implementation on decompressive hemicraniectomy (DH) in Germany, accounting for the effects of the COVID-19 pandemic. **Methods**: Annual treatment numbers (2015 to 2023) for IVT, MT, DH, cranioplasty (CP), and Computer-Aided Design CP (CAD CP) were extracted using the Operation and Procedure Codes (OPS). Age and sex distributions were analysed in four age groups (0–39, 40–59, 60–79, ≥80 years). Hospitalizations for AIS, subarachnoid haemorrhage (SAH), and intracranial haemorrhage (ICH) were included. Annual treatment rates were calculated. **Results**: In 2015 250,802 patients with AIS, 11,082 patients with SAH and 47,336 patients with ICH were documented. Overall, AIS hospitalizations declined slightly by 1.1%, whereas SAH and ICH decreased more markedly by 15.6% and 14.3%, respectively, with the most pronounced reductions observed during the COVID-19 pandemic. Among AIS patients, intravenous thrombolysis increased from 16% to 20%, while mechanical thrombectomy rates increased from 3% to 9%. Both reperfusion therapies showed increasing use particularly among patients aged ≥80 years (IVT: +35%; MT: +42%). DH remained overall stable (+2.82%), with a predominance in men (60%). In contrast, CP procedures declined by 25.3%, whereas CAD CP increased by 35% during the observation period. **Conclusions**: Long-term increasing trends in reperfusion therapies, especially mechanical thrombectomy, were largely unaffected by the COVID-19 pandemic and—contrary to expectation—obviously had no influence on the emergence of decompressive hemicraniectomy in our descriptive analysis.

## 1. Introduction

Before a new therapeutic procedure enters the clinical application, numerous mandated clinical trials are conducted. These trials not only evaluate the efficacy of the intervention but also systematically assess its associated risks. The implementation of a new therapy into routine care can therefore have relevant consequences, including effects on mortality, morbidity, and the incidence of related complications [1].

The introduction of intravenous thrombolysis (IVT) and, more recently, mechanical thrombectomy (MT) for acute ischemic stroke (AIS) represents notable examples in this context. Both have substantially improved patient outcomes, while also altering the epidemiology of stroke-related complications [2]. Similar shifts have been observed with other innovations, such as the impact of IVT on rates of intracerebral haemorrhage (ICH), or the implementation of new oral anticoagulants (NOACs) in secondary prevention [3,4].

The integration of new procedures into clinical practice typically follows a temporal trajectory. Structural adjustments within healthcare systems are often necessary. As a result, treatment rates typically rise gradually at first and eventually level off once nearly all eligible patients have been treated. In parallel, the frequency of associated complications may rise or decline [5].

With the paradigm shift introduced by MT in 2015, it could be hypothesized that early and effective reperfusion could limit infarct size, reduce the development of malignant oedema and consequently decrease the need for decompressive hemicraniectomy (DH). In line with this hypothesis, a recent study from the United States reported a decline in DH rates following the widespread adoption of MT [6]. A single-centre study from Germany evaluated the impact of landmark randomized controlled trials, including DESTINY [7] and MR CLEAN [8], on DH rates for acute ischemic stroke between 2000 and 2016. Despite the introduction of these trials, the authors reported a decline in DH procedures after 2014 [9]. However, external factors can further modulate treatment rates and disease incidence, and the reported data refers to preselected patient groups. The recent global COVID-19 pandemic demonstrated this vividly, with multiple studies documenting striking shifts in hospital admissions, complication rates, and therapeutic utilization [10].

With regard to this background, the present study aims to investigate the impact of the implementation of mechanical thrombectomy for acute ischemic stroke on the frequency of decompressive hemicraniectomies documented in a nationwide mandatory registry in Germany. A particular focus of this analysis was the consideration of the COVID-19 pandemic, as clear evidence supporting the benefit of MT emerged in 2015 and its subsequent integration into routine practice inevitably overlapped with the pandemic period.

## 2. Methods

### 2.1. Database

For this purpose, we conducted a retrospective analysis using the German Diagnosis-Related Group (DRG, Statistisches Bundesamt, Wiesbaden, Germany) statistics from 2015 to 2023. Treatment numbers were analysed annually based on the Operation and Procedure Codes (OPS) for intravenous thrombolysis (8-020.8), mechanical thrombectomy (8-836.80), decompressive hemicraniectomy (5-012.0), cranioplasty (5-020.2) and Computer-Aided Design cranioplasty (5-020.71-75). This coding system is publicly accessible at www.icd-code.de (accessed on 27 September 2025). Additionally, we examined the age and sex distribution of patients undergoing these treatments. Age groups were categorized as follows: 0–39 years, 40–59 years, 60–79 years, and 80 years and older. Sex distribution was divided into female and male patients.

Furthermore, we analysed all hospital cases coded with ICD-10 code I60 for subarachnoid haemorrhage, I61 for intracranial haemorrhage, I62 for non-traumatic intracranial haemorrhage and I63 for acute ischemic stroke. I61 and I62 were added together, and no distinction was made between these two codes for the graphs. Data were obtained via the DESTATIS website [11]. Based on these data, we calculated annual treatment rates for IVT, MT, DH, CP and CAD CP by comparing the number of patients receiving these interventions to the total number of ischemic stroke hospitalizations each year or haemorrhage hospitalizations.

As this study analysed in an anonymous format exclusively retrospective collected data, no informed consent from the patients was necessary or available. Based on German law, anonymous electronic medical data can be used for research, provided certain conditions are met. This legislation allows the use of these de-identified records without obtaining written informed consent from the patients or approval from a medical ethics committee.

### 2.2. Study Population

The study sample included a total of 2,153,495 hospitalizations for AIS (ICD-10 code I63), 93,091 hospitalizations for SAH (ICD-10 code I60) and 391,913 hospitalizations for ICH (ICD-10 codes I61 and I62) in Germany between 2015 and 2023. Among these, 394,571 patients received IVT, 172,841 underwent MT, 35,578 underwent DH, 23,597 received CP and 9721 underwent CAD-CP as identified by the OPS.

### 2.3. Statistical Analyses

The statistical analysis was restricted to descriptive approaches, and no inferential statistics were applied. To assess trends over time, we calculated absolute frequencies and annual treatment rates for IVT, MT, DH, CP and CAD CP among patients with AIS, SAH, traumatic ICH and non-traumatic ICH in Germany between 2015 and 2023. Time trends were visualized using line graphs for annual IVT, MT, DH, CP and CAD CP rates, as well as age- and sex-specific subgroup analyses.

## 3. Results

### 3.1. Incidences over Time

**Figure 1:**
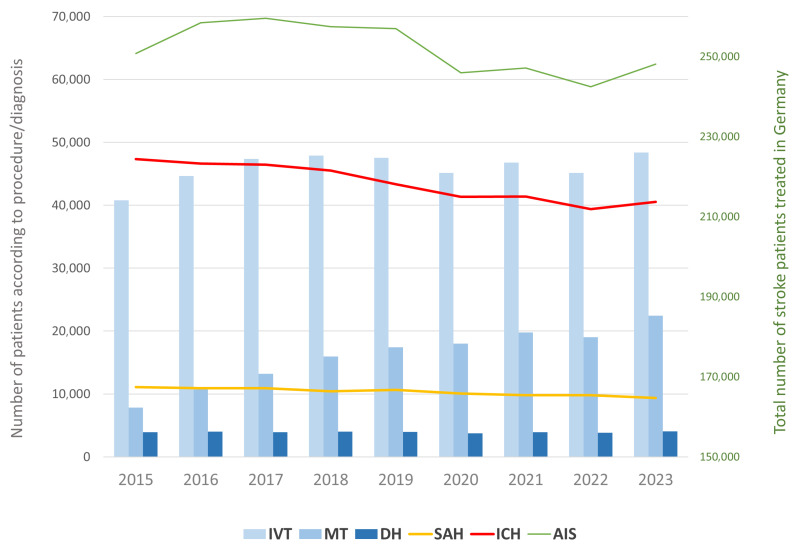
Trends in subarachnoid haemorrhage (SAH), intracerebral haemorrhage (ICH) (non-traumatic and traumatic) and acute ischemic stroke (AIS) cases and treatment with intravenous thrombolysis (IVT), mechanical thrombectomy (MT) and decompressive hemicraniectomy (DH) in Germany from 2015 to 2023. The bar chart displays the absolute number of patients treated with IVT (light blue), MT (blue) and DH (dark blue). The line graphs show the total number of SAH cases (yellow), ICH cases (red) and AIS cases (green) recorded annually. The AIS curve is referred to on the y-axis right; the other parameters are referred to on the y-axis left. IVT = intravenous thrombolysis, MT = mechanical thrombectomy, DH = decompressive hemicraniectomy, SAH = subarachnoid haemorrhage, ICH = intracerebral haemorrhage, AIS = acute ischemic stroke.

In 2015, a total of 250,802 patients with AIS were documented in Germany. By 2023, this number had slightly declined to 248,107, representing a 1.1% overall decrease. The most pronounced reduction occurred during the COVID-19 pandemic, with a 4.3% drop observed between 2019 and 2020 (Figure 1). In 2015, a total of 11,082 patients with SAH were documented. By 2023, this number had declined to 9350, representing a 15.6% overall decrease. The most pronounced reduction for SAH also occurred during the COVID-19 pandemic, with a 5.4% drop observed between 2019 and 2020. In 2015, a total of 47,336 patients with traumatic and non-traumatic ICH were documented in Germany. By 2023, this number had slightly declined to 40,560, representing a 14.3% overall decrease. The most pronounced reduction for ICH, with a 4.8% drop, was observed between 2018 and 2019 (Figure 1 and Table 1).

### 3.2. Intravenous Thrombolysis Trends

The use of IVT demonstrated a continuous increase in both absolute numbers and relative proportions over the study period. In 2015, 40,766 patients with AIS (16.25%) received IVT, compared with 48,378 patients (19.50%) in 2023 (Figure 1). Age stratification showed a rising proportion of elderly patients, with the share of IVT-treated individuals aged ≥80 years increasing from 30.8% in 2015 to 35.3% in 2023.

### 3.3. Mechanical Thrombectomy Trends

MT showed a marked upward trend: in 2015, 7840 AIS patients (3.13%) underwent MT, compared with 22,445 patients (9.05%) in 2023, representing nearly a threefold increase (Figure 1). This increase was consistent throughout the entire observation period. The proportion of patients aged ≥80 years undergoing MT rose from 27.2% in 2015 to 42.1% in 2023.

### 3.4. Decompressive Hemicraniectomy Trends

The use of DH has remained constant over the years (2015–2023). In 2015, 3933 patients underwent DH compared to 4044 patients in 2023 (+2.82%) (Figure 1). Across the entire study period, procedures were more frequently performed in men than in women (60% vs. 40%). No consistent temporal trends were observed across age groups. The majority of patients undergoing DH were between 40 and 79 years old (70–80%), while 17–20% were younger than 39 years and 6–9% were older than 80 years. Women, on average, were slightly older.

### 3.5. Cranioplasty Trend

**Figure 2:**
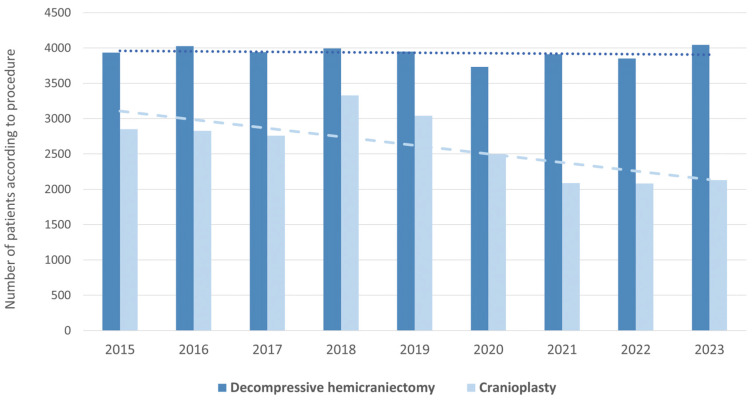
Trends in decompressive hemicraniectomy (DH) and cranioplasty (CP) in Germany from 2015 to 2023. The bar chart displays the number of patients treated with DH (dark blue) and CP (light blue) recorded annually. DH = decompressive hemicraniectomy, CP = cranioplasty.

**Figure 3:**
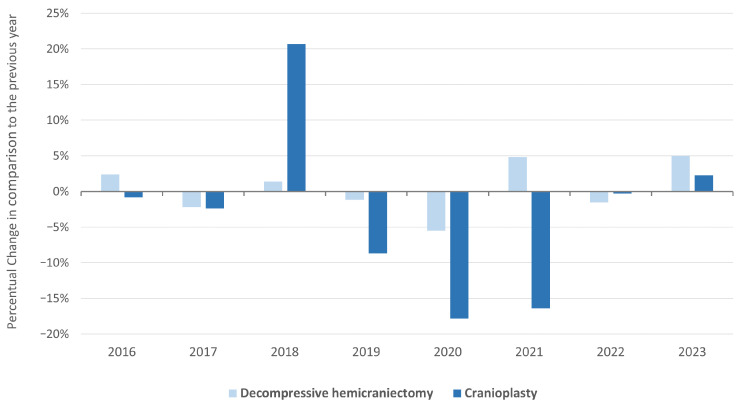
Percentage changes in decompressive hemicraniectomy (DH) and cranioplasty (CP) in Germany from 2015 to 2023. The bar chart displays the percentage changes in DH (light blue) and CP (dark blue) in comparison to absolute numbers documented the previous year. DH = decompressive hemicraniectomy, CP = cranioplasty.

CP procedures showed a clear decline over the observation period (Figure 2). In 2015, 2850 patients underwent CP, compared with 2129 in 2023, representing a reduction of 25.3%. The highest reduction, of 18%, was shown during the pandemic period in 2020, followed by a reduction of 16% in 2021, also during the pandemic period (Figure 3). Throughout the study period, men accounted for the majority of procedures (55%). However, the overall number of CPs performed in men decreased by 22.2%. Women, in contrast, were on average slightly older at the time of CP. Notably, the decrease in procedures was even more pronounced in women, with a reduction of 29.3% between 2015 and 2023.

### 3.6. Computer-Aided Design Cranioplasty

**Figure 4:**
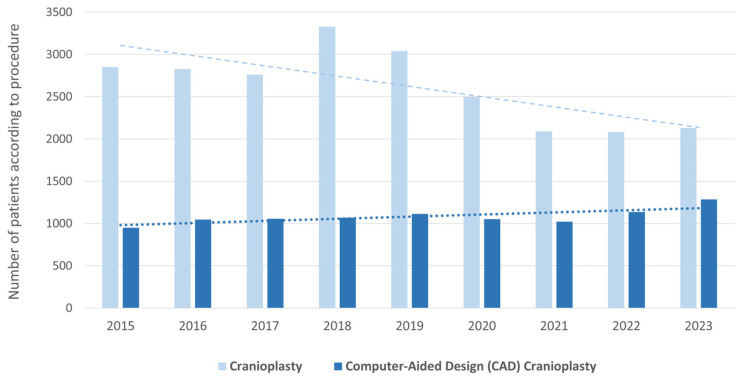
Trends in cranioplasty (CP) and Computer-Aided Design (CAD) cranioplasty (CAD CP) in Germany from 2015 to 2023. The bar chart displays the absolute number of patients treated with CP (dark blue) and CAD CP (light blue) recorded annually. CP = cranioplasty, CAD CP = Computer-Aided Design cranioplasty.

CAD CP rates increased over the years; in 2015, 950 patients underwent CAD CP, in addition to 1284 patients in 2023 (+35.16%) (Figure 4). There was a slight decrease during the COVID-19 pandemic in 2020 and 2021 (−5.58%; −2.67%). Over the years, more men than women underwent CAD CP (55% to 45%). However, the overall number of CAD CPs performed in men increased by 27.35%, while the overall number performed in women decreased by 6.50%.

## 4. Discussion

### 4.1. Overview of Findings

This nationwide analysis of German hospital data from 2015 to 2023 revealed distinct trends across major cerebrovascular procedures. The overall number of patients hospitalized with acute AIS showed a slight decline from 2015 to 2023 (−1.1%), with the most pronounced reduction during the COVID-19 pandemic between 2019 and 2020 (−4.3%). Similar pandemic-related decreases were also observed for SAH (−15.6% overall) and ICH (−14.3% overall).

The utilization of reperfusion therapies increased substantially. IVT rose from 16.3% in 2015 to 19.5% in 2023. The expansion was even more striking for MT, which nearly tripled from 7840 procedures (3.1%) in 2015 to 22,445 (9.1%) in 2023, reflecting the rapid nationwide adoption of this evidence-based procedure.

In contrast to these upward trends, DH remained largely unchanged over time, with 3933 procedures in 2015 and 4044 in 2023 (+2.8%), and consistent sex (60% men, 40% women) and age distributions. CP procedures declined markedly (−25.3%) over the period of observation (2015–2023), with the most pronounced reductions during the pandemic years 2020 and 2021. The decline was more pronounced among women (−29.3%) than men (−22.2%). In contrast, CAD CP showed a continuous increase, from 950 procedures in 2015 to 1284 in 2023 (+35.2%), despite temporary decreases during the pandemic years.

### 4.2. Demographic Shifts in Treatment Patterns

A previous paper has shown that the increase in the use of IVT and MT in recent years has been driven primarily by patients aged 80 years and older [12]. Our findings support this observation: both IVT and MT demonstrated a steady rise among elderly stroke patients throughout the study period. This trend may reflect growing evidence supporting the safety and efficacy of reperfusion therapies in octogenarians when guided by appropriate selection criteria and modern imaging, but it could also partly result from a learning-curve effect as physicians gain experience in routine practice. Interestingly, the most pronounced increase in MT procedures was observed among women aged 80 years and older.

The present analysis revealed distinct age- and sex-related patterns across all examined procedures. Men underwent DH more frequently than women, with a consistent male predominance of approximately 60%. This finding is in line with previous reports demonstrating that men tend to experience cerebrovascular diseases at a younger age than women, likely due to differences in vascular risk profiles and the protective effect of oestrogens in premenopausal women [13]. Consequently, the higher DH rate among men may reflect their earlier onset of severe ischemic events requiring surgical intervention.

In contrast, women in our study were on average slightly older at the time of intervention, particularly in DH and CP procedures. This aligns with epidemiological data suggesting that female patients often present with stroke at a later age, but may experience worse outcomes once a severe event occurs [14]. The relatively stable age distribution across the study years indicates that these sex-specific differences have remained consistent over time, despite the overall evolution of acute stroke management and the increasing availability of endovascular treatments. In contrast to previous papers, our nationwide analysis did not demonstrate a decline in DH rates following the introduction of mechanical thrombectomy [5,8]. Our study shows a completely contrary trend in this regard; over time, we observe no noteworthy change in the number of DHs performed. The analysis of our data may also be influenced by potential biases; however, in this context, our evaluation includes data from a national mandatory registry in which, according to current reports, nearly all of the procedures performed nationwide should be documented [15]. However, women underwent CP less often than men (45% to 55%), and the rate decreased by nearly 30% over the years. Among men, the overall rate declined by approximately 22%. Overall, it can be speculated that improvements in medical care—on the one hand, mechanical thrombectomy, and on the other hand, advances in neurocritical care—may have contributed to the overall stable trend in DH rates observed in our study. Due to improvements in neurocritical care, more critically ill patients may survive and subsequently require decompressive hemicraniectomy (DH). Conversely, the number of patients with malignant middle cerebral artery infarction may have decreased due to the more frequent use of mechanical thrombectomy.

An additional noteworthy finding was the trend observed in CAD CP. The number of CAD CP procedures increased by about 35% over the study period. When stratified by sex, procedures in men rose by 27%, whereas those in women declined by 6%. The observed decline in CP (−22% among men) may reflect a more selective indication process in recent years, particularly for patients with higher comorbidity or limited neurological recovery. In addition, the COVID-19 pandemic likely contributed to a temporary reduction in elective procedures, which may have disproportionately affected male patients. In line with previous investigations, our study showed a reduction in acute strokes during the pandemic [10]. This effect manifested as a slight downward shift in the use of intravenous thrombolysis. For intracerebral haemorrhage, the pandemic slightly accentuated the pre-existing downward trend. In contrast, no deviation during the pandemic was evident for the procedures of mechanical thrombectomy or decompressive hemicraniectomy. While, in the context of ischemic stroke, a number of stroke mimics and transient ischemic attacks likely did not seek medical care, this pandemic-related effect was not observed for other entities/procedures, which, due to their severity, become clinically apparent and are therefore brought to medical attention. The same explanation may also account for the unchanged use of decompressive hemicraniectomy during the pandemic.

In contrast, the marked rise in CAD CP (+35% overall) suggests broader adoption of advanced reconstructive technologies in clinical practice. CAD-based implants offer improved precision, reduced operative time, and favourable cosmetic outcomes, making them increasingly preferred over conventional methods [16]. The stronger increase among men (+27%) may correspond to their higher rate of prior decompressive surgeries, while the slight decline among women (−6%) could relate to demographic factors or smaller case volumes.

Overall, these findings illustrate an ongoing shift toward more individualized and technology-driven cranial reconstruction, alongside subtle age- and sex-related differences in procedural application.

### 4.3. Expanded Indications and Evolving Clinical Practice

Existing recommendations, primarily from the United Kingdom and Canada, address DH in malignant middle cerebral artery infarction but do not provide standardized criteria for patient selection or timing [17]. In clinical practice, DH is sometimes performed pre-emptively, a strategy supported by landmark trials such as DESTINY, which demonstrated significant mortality reduction with early intervention [7]. This lack of standardized guidance may partly explain the stable DH rates observed in our analysis, as clinical decisions remain largely experience-driven and centre-specific. Future consensus guidelines could help harmonize indications, optimize patient selection, and ultimately improve outcome comparability across institutions.

### 4.4. Limitations

This study has several limitations. First, the analysis is based on administrative data, which may be subject to coding inconsistencies and lacks clinical detail such as stroke severity, symptom onset times, infarct size, timing of MT/DH, or functional outcomes. Second, behavioural and environmental risk factors (e.g., smoking, alcohol consumption, physical activity) were not available, precluding analysis of their potential influence. Third, the effects of regional variability and differences in hospital resources could not be assessed. The vagueness in (non-) traumatic ICH may represent a fourth drawback. At this point, another aspect that needs to be mentioned is the data basis of our analysis. Our study is based exclusively on aggregated data, and individual-level information was not available. Furthermore, due to the data, it was not possible to perform adjustment for confounders or an analysis of outcome data. In addition, the influence of a potential coding bias cannot be estimated. When interpreting our results, the ecological nature of the study must therefore be taken into account. Finally, the descriptive nature of the study limits causal inference.

### 4.5. Strengths

Despite these limitations, this study benefits from the use of comprehensive national data, covering nearly all hospital-based AIS, ICH and SAH cases in Germany over a nine-year period. The large sample size and consistent documentation provide robust insights into real-world treatment patterns. These findings can serve as a valuable reference for health system evaluation and future stroke and haemorrhage care planning.

## 5. Conclusions

Our findings demonstrate an evolving development in the treatment and management of acute ischaemic stroke in Germany, with sustained expansion of reperfusion therapies, including mechanical thrombectomy and intravenous thrombolysis. Nevertheless, according to our descriptive analysis, this increase obviously has not influenced the use of decompressive surgery. Although it might be anticipated that wider adoption of mechanical thrombectomy, by lowering the incidence of severe infarcts, would translate into fewer decompressive hemicraniectomies, our data revealed that the utilization of this procedure remained largely unchanged over time. It may be speculated that the benefits of thrombectomy do not substantially affect the likelihood of requiring decompressive surgery at a later stage. Our findings contrast with previously published data on this topic. The extent to which the trend we observed can be generalised remains speculative; however, it should be noted that, unlike earlier reports, our results are based on comprehensive, nationwide data from a country such as Germany. The partial decline in cranioplasty (overall −25%) while simultaneously increasing utilisation of Computer-Aided Design cranioplasty (+35%) might be a result of favourable safety patterns for Computer-Aided Design cranioplasty. The COVID-19 pandemic determined an overall temporary decrease in all entities and procedures, without influencing long-term trends. Comparable investigations will also be required in the future to identify correlations and, in particular, to recognize potential mis-developments.

## Figures and Tables

**Table 1:** Absolute frequencies of selected entities recorded in a nationwide mandatory registry in Germany, 2015–2023.

ICD/OPS	2015	2016	2017	2018	2019	2020	2021	2022	2023
I60 SAH	11,082	10,907	10,935	10,423	10,667	10,090	9823	9814	9350
I61 & I62 ICH	47,336	46,633	46,433	45,507	43,334	41,333	41,373	39,404	40,560
I63 AIS	250,802	258,480	259,594	257,472	256,965	245,944	247,176	242,492	248,107
IVT (8-020.8)	40,766	44,664	47,366	47,906	47,553	45,149	46,772	45,148	48,378
MT (8-836.80)	7840	10,693	13,203	15,956	17,423	17,989	19,766	19,040	22,445
DH (5-012.0)	3933	4027	3939	3994	3948	3731	3911	3851	4044
CP (5-020.2)	2850	2826	2758	3328	3039	2497	2088	2 082	2129
CAD CP (5-020.71-75)	950	1045	1056	1068	1112	1050	1022	1134	1284

SAH = subarachnoid haemorrhage. ICH = intracerebral haemorrhage. AIS = acute ischaemic stroke. IVT = intravenous thrombolysis. MT = mechanical thrombectomy. DH = decompressive hemicraniectomy. CP = cranioplasty. CAD CP = Computer-Aided Design CP.

## Data Availability

The original contributions presented in this study are included in the article. Further inquiries can be directed to the corresponding author.
